# Oncogenic Ras mutant causes the hyperactivation of NF‐κB via acceleration of its transcriptional activation

**DOI:** 10.1002/1878-0261.12580

**Published:** 2019-10-18

**Authors:** Kenji Tago, Megumi Funakoshi‐Tago, Satoshi Ohta, Hirotoshi Kawata, Hiroshi Saitoh, Hisanaga Horie, Chihiro Aoki‐Ohmura, Junji Yamauchi, Akira Tanaka, Jitsuhiro Matsugi, Ken Yanagisawa

**Affiliations:** ^1^ Division of Structural Biochemistry Department of Biochemistry Jichi Medical University Shimotsuke Japan; ^2^ Division of Hygienic Chemistry Faculty of Pharmacy Keio University Minato‐ku Japan; ^3^ Department of Pathology Jichi Medical University Shimotsuke Japan; ^4^ Department of Surgery Jichi Medical University Shimotsuke Japan; ^5^ Laboratory of Molecular Neuroscience and Neurology Tokyo University of Pharmacy and Life Sciences Hachioji Japan

**Keywords:** colorectal cancer, MSK1/2, NF‐κB, p65/RelA, Ras

## Abstract

It is well established that nuclear factor κB (NF‐κB) acts as one of the most important transcription factors for tumor initiation and progression, as it both protects cells from apoptotic/necrotic signals and accelerates angiogenesis and tumor metastasis, which is mediated via the expression of target genes. However, it has not yet been clarified how oncogenic signals accelerate the activation of NF‐κB. In the current study, we utilized untransformed NIH‐3T3 cells stably harboring a κB‐driven luciferase gene to show that an oncogenic mutant of Ras GTPase augmented TNFα‐induced NF‐κB activation. Notably, enforced expression of cyclin‐dependent kinase inhibitors, such as p27^Kip1^ and p21^Cip1^, effectively canceled the accelerated activation of NF‐κB, suggesting that oncogenic Ras‐induced cell cycle progression is essential for the hyperactivation of NF‐κB. Furthermore, we found that Ras (G12V) augmented the transcriptional activation of NF‐κB, and this activation required the p38 MAP kinase. We observed that a downstream kinase of p38 MAP kinase, MSK1, was activated by Ras (G12V) and catalyzed the phosphorylation of p65/RelA at Ser‐276, which is critical for its transcriptional activation. Significantly, phosphorylation of the p65/RelA subunit at Ser‐276 was elevated in patient samples of colorectal cancer harboring oncogenic mutations of the K‐Ras gene, and the expression levels of NF‐κB target genes were drastically enhanced in several cancer tissues. These observations strongly suggest that oncogenic signal‐induced acceleration of NF‐κB activation is caused by activation of the p38 MAP kinase–MSK1 signaling axis and by cell cycle progression in cancer cells.

**Abbreviations**
CDK: cyclin‐dependent kinase
CHX: cycloheximide
COX‐2: cyclooxygenase 2
CRC: colorectal cancer
DMEM: Dulbecco's modified Eagle's medium
GAP: GTPase‐activating protein
ICAM‐1: intercellular adhesion molecule‐1
IκBα: inhibitor of κBα
MSK1/2: mitogen and stress activated protein kinase 1/2
NF‐κB: nuclear factor κB
NPM: nucleophosmin
PI‐3 kinase: phosphatidylinositol‐3 kinase
PKA: protein kinase A
RHD: Rel homology domain
shRNA: short‐hairpin RNA
TNFα: tumor necrosis factor α
VEGF: vascular endothelial growth factor

## 1 Introduction

Nuclear factor κB (NF‐κB) includes a family of transcription factors, which induce the expression of a number of target genes related to anti‐inflammation, development, and the apoptosis‐related responses (Baud and Karin, 2009; Ghosh and Hayden, 2008). Mammalian NF‐κB subunits are classified as five molecules: p65/RelA, RelB, c‐Rel, p50, and p52. It is well understood that p50 and p52 are produced by proteolysis of their precursor proteins, NF‐κB1 p105 and NF‐κB2 p100, respectively. These five NF‐κB family members possess a Rel homology domain (RHD) contributing to homo‐ and heterodimerization, nuclear translocation, and interaction with inhibitor proteins such as IκB family (Baldwin, 1996; Ghosh and Karin, 2002). Among five NF‐κB members, p65/RelA, RelB, and c‐Rel contain the transcriptional activation domain in their carboxy terminus. The transcriptional activity of NF‐κB family requires the formation of homo‐ or heterodimeric protein complexes, such as p65/RelA·p50 and p50·p50. Under unstimulated condition, NF‐κB family is known to be negatively regulated by IκB family. IκB family includes five members such as IκBα, IκBβ, IκBγ, IκBε, and Bcl‐3, which possess ankyrin repeat motif contributing to the interaction with NF‐κB. In addition, NF‐κB1 p105 and NF‐κB2 p100 are also included in IκB family because of the presence of ankyrin repeat motif. IκB proteins interact with NF‐κB dimers and block their nuclear localization, thereby resulting in their retention in cytoplasm. Once cells are stimulated with various cytokines, such as TNFα, IL‐1, and CD40 ligand, the phosphorylation of IκB proteins is induced and this triggers ubiquitin‐proteasomal degradation of IκB proteins. Then, the released NF‐κB, such as p50·p65/RelA heterodimer, is translocated into the nucleus and binds to κB‐responsive element of their target genes. Furthermore, to exhibit the transcriptional activity of NF‐κB, a direct interaction with the transcriptional coactivator p300/CBP is required (Zhong *et al.*, 1998).

It was also reported that NF‐κB contributes to tumor progression by accelerating the expression of diverse target genes, which contribute to cell proliferation, angiogenesis, and metastasis. Cyclin D1 and c‐Myc are reported as target genes of NF‐κB, which is understood to play important roles in cell proliferation and tumorigenesis (Guttridge *et al.*, 1999; Kang *et al.*, 2015). During tumor progression, angiogenesis and metastasis are critical events. Angiogenesis is accelerated by several growth factors and cytokines, such as vascular endothelial growth factor (VEGF), interleukin‐6 (IL‐6) and IL‐8, and their expression is directly or indirectly enhanced by NF‐κB activation (Libermann and Baltimore, 1990; Richmond, 2002). Additionally, for metastasis, NF‐κB was reported to induce the expression of MMP9 and cyclooxygenase 2 (COX‐2), which contributed to the migration and invasion of cancer cells (Choo *et al.*, 2008; Tsujii *et al.*, 1998). Indeed, the inhibition of tumor progression caused by inhibitors against NF‐κB signals has been shown in colon, lung, and breast cancers, whereas it is still unclear how the activity of NF‐κB is regulated by oncogenic signals (Fernandez‐Majada *et al.*, 2007; Rahman *et al.*, 2007).

Small GTPases such as Ras family possess the activities to bind to GTP and hydrolysis GTP to GDP, and GTP‐bound form of Ras family exhibits their biological activities, such as interacting with their target effectors and regulating their activities (Kaziro *et al.*, 1991). Ras family include three classical member, H‐Ras, K‐Ras, and N‐Ras, and these three proteins function as critical GTPases involved in cell proliferation, cell survival, and development in a wide variety of mammalian cells (Satoh *et al.*, 1992b). Activation of Ras proteins is triggered by stimulation with various growth factors and cytokines, including EGF, PDGF, erythropoietin, and IL‐2, and these stimulations accelerate GDP‐GTP exchange reaction on Ras proteins mediated by guanine nucleotide exchange factor, such as Son of Sevenless and RasGRFs. Once activated, Ras proteins bind and activate their downstream molecules such as Raf kinases, phosphatidylinositol‐3 kinase (PI‐3 kinase), and phospholipase C‐ε (Rodriguez‐Viciana *et al.*, 2005; Stephen *et al.*, 2014). Subsequently, the biological activities of Ras proteins are shut off by their own GTP‐hydrolysis activity, and this step is accelerated by GTPase‐activating protein (GAP) including p120GAP, NF1, and Gap1m (Satoh *et al.*, 1990, 1992a; Torti *et al.*, 1992). In a number of tumors, point mutation of Ras gene, especially K‐Ras gene such as G12V and Q61L, is found. These point mutations cause constitutive activation of Ras proteins by loss of their GTPase activity, and the activation of downstream of Ras proteins is drastically augmented (Bos, 1989; Karnoub and Weinberg, 2008). However, it is still unclear how these signaling molecules contribute to cellular transformation and tumor progression.

In the current study, we found that an oncogenic mutant of Ras augments cytokine‐induced NF‐κB activation. We clarified part of the molecular mechanism by which oncogenic Ras mutant‐induced signals accelerate the activation of NF‐κB.

## 2 Materials and methods

### 2.1 Cell culture

For the culture of cell lines such as human lung cancer cell line A549, HEK293T cells, and murine fibroblast, NIH‐3T3 cells, Dulbecco's modified Eagle's medium (DMEM) supplemented with 10% FBS, 2 mm glutamine, 100 units·mL^−1^ penicillin, and 100 μg·mL^−1^ streptomycin was utilized. A subline of NIH‐3T3 cells, KF‐8 stably possessing κB‐luciferase genes, was cultured in complete DMEM supplemented with 7.5 μg·mL^−1^ puromycin (Tago *et al.*, 2010).

### 2.2 Plasmids construction

pBabePuro‐H‐Ras (G12V) was a kind gift from S. Lowe, Sloan Kettering Institute. pBabePuro‐B‐RAF (V600E) was a gift from W. Hahn (Addgene plasmid # 15269). cDNA of p16Ink4a, p27^Kip1^, and p21^Cip1^ were amplified by PCR using PrimeSTAR GXL DNA polymerase (TAKARA‐Bio, Shiga, Japan) and inserted into *Eco*RI and *Xho*I sites of MSCV‐ires‐Puro retroviral vector.

### 2.3 Antibodies and chemicals

An anti‐FLAG antibody (M2) was purchased from Merck Millipore (St. Charles, MO, USA). Anti‐cyclin A (SC‐751), anti‐cyclin D1 (SC‐753), anti‐CDC2 (SC‐163), anti‐CDK4 (SC‐601), anti‐H‐Ras (SC‐520), and anti‐β‐actin (SC‐130301) antibodies were obtained from Santa Cruz Biotechnology (Dallas, TX, USA). An anti‐phospho p65/RelA (S276) antibody was purchased from Rockland Immunochemicals (Limerick, PA, USA). Anti‐MSK1 and anti‐MSK2 antibodies were purchased from Cell Signaling Technology (#9309; Danvers, MA, USA) and BD Biosciences (San Jose, CA, USA), respectively. TNFα and PDGF were purchased from PeproTech (Rocky Hill, NJ, USA). LY294002, U0126, SP600125, and SB203580 were obtained from FUJIFILM Wako Pure Chemical (Osaka, Japan). NOX inhibitor, VAS2870, was purchased from Merck Millipore (Burlington, MA, USA).

### 2.4 Retrovirus production and infection

To obtain retroviruses, MSCV‐ires‐GFP or MSCV‐ires‐Puro encoding the cDNA of the indicated proteins was transfected into HEK293T cells (1 × 10^6^ cells per 60‐mm‐diameter culture dish) with helpers such as pE‐Eco and pGP (TAKARA‐Bio). Twenty‐four hours later, culture medium was replaced with 1.5 mL fresh culture medium. Secreted retroviruses were harvested every 4–6 h during 24–60 h post‐transfection, pooled, and stored on ice. Exponentially growing cells (1 × 10^5^ cells per 60‐mm‐diameter culture dish) were infected several times at 2‐h intervals with 2 mL of virus‐containing conditioned medium with 1.0 µg·mL^−1^ polybrene (Merck Millipore). Twenty‐four hours later, the infected cells were cultured in completed medium including suitable concentrations of puromycin (1.5 μg·mL^−1^ for A549, and 7.5 μg·mL^−1^ for NIH‐3T3 cells) for 3 days. Then, selected cells were utilized for the experiments.

### 2.5 RNA interference for MSK1/2 and K‐Ras

Annealed 60‐mer oligonucleotides including the sequences of a short‐hairpin RNA (shRNA) against murine MSK1/2 and human K‐Ras gene products were inserted into pSUPER‐retro‐puro retroviral plasmid (Oligoengine, Seattle, WA, USA). The sequences of oligonucleotides used for constructing the shRNA retroviral vector against each gene product were described as below: For sh‐mMSK1#1; GATCCCCGTGATTTACCAGAGAGAAATTCAAGAGATTTCTCTCTGGTAAATCACTTTTTA and AGCTTAAAAAGTGATTTACCAGAGAGAAATCTCTTGAATTTCTCTCTGGTAAATCACGGG, For sh‐mMSK1#2; GATCCCCGCCAATACTCAGAAAGAAATTCAAGAGATTTCTTTCTGAGTATTGGCTTTTTA and AGCTTAAAAAGCCAATACTCAGAAAGAAATCTCTTGAATTTCTTTCTGAGTATTGGCGGG, For sh‐MSK2#1; GATCCCCGCGGAGAGCTATTGGAACATTCAAGAGATGTTCCAATAGCTCTCCGCTTTTTA and AGCTTAAAAAGCGGAGAGCTATTGGAACATCTCTTGAATGTTCCAATAGCTCTCCGCGGG, For sh‐MSK2#2; GATCCCCGGGCATGAGGAGAAGGTGATTCAAGAGATCACCTTCTCCTCATGCCCTTTTTA and AGCTTAAAAAGGGCATGAGGAGAAGGTGATCTCTTGAATCACCTTCTCCTCATGCCCGGG, For sh‐K‐Ras#1; GATCCCCCGAATATGATCCAACAATATTCAAGAGATATTGTTGGATCATATTCGTTTTTA and AGCTTAAAAACGAATATGATCCAACAATATCTCTTGAATATTGTTGGATCATATTCGGGG, For sh‐K‐Ras#2; GATCCCCGGACGAATATGATCCAACATTCAAGAGATGTTGGATCATATTCGTCCTTTTTA and AGCTTAAAAAGGACGAATATGATCCAACATCTCTTGAATGTTGGATCATATTCGTCCGGG.

Infection with retroviruses, including these shRNA, into NIH‐3T3 cells was performed at the same time with a retrovirus harboring oncogenic Ras.

### 2.6 Reporter gene analysis

KF‐8 cells infected with indicated retroviruses were re‐plated on a 24‐well plate and then cultured in serum‐starved medium for 24 h. Then, the cells were stimulated with 10 ng·mL^−1^ TNFα for 16 h. Cells were lysed with passive lysis buffer (Promega, Madison, WI, USA), and the activity of expressed luciferase was measured by using a Luciferase Assay kit (Promega). The specific activity of luciferase was calculated by normalizing with protein concentration of each cell lysate, and data were shown in the graph as relative amount. To observe the effect of each kinase inhibitor on the NF‐κB activation, cells were treated with the inhibitors 15 min prior to stimulation with TNFα. To analyze the transcriptional activation of NF‐κB, parental NIH‐3T3 cells were transfected with pFR‐luciferase containing the sequence of a GAL4‐responsive element with the indicated combination of plasmids. Cells were cultured in serum‐free DMEM for 24 h, and then, the cells were harvested, and luciferase activity was assayed as described above.

### 2.7 DNA pull‐down assay

Nuclear extracts were prepared as reported previously (Dignam *et al.*, 1983). Nuclear extracts were incubated with a biotin‐conjugated κB‐binding element in the presence of 20 mm HEPES–KOH pH7.5 including 1 μg·μL^−1^ poly‐dIdC for 30 min at 25 °C. Then, the binding mixtures were pulled down using streptavidin‐conjugated agarose (FUJIFILM Wako Pure Chemical). The samples were analyzed by immunoblotting analysis as described below.

### 2.8 *In vitro* kinase assay

HEK293T cells were transfected with the indicated combinations of plasmids harboring cDNA of FLAG‐MSK1, FLAG‐PKAc, or H‐Ras (G12V). Twenty‐four hours later, the transfected cells were cultured in serum‐free DMEM for 24 h. Then, cells were harvested, and cell lysates were prepared with Nonidet P‐40 (NP‐40) lysis buffer (50 mm Tris/HCl pH 7.4, 10% glycerol, 50 mm NaCl, 0.5% sodium deoxycholate, 0.5% NP‐40, 20 mm NaF, and 0.2 mm Na_3_VO_4_) supplemented with protease inhibitors. FLAG‐MSK1 and FLAG‐PKAc were purified by immunoprecipitation using an M2 antibody attached to protein G‐sepharose (GE Healthcare, Chicago, IL, USA), and they were utilized for *in vitro* kinase assays. For these assays, purified kinases were incubated with 2 μg GST‐p65/RelA and 100 μm ATP for 30 min at 30 °C. The reaction was terminated by the addition of Laemmli sample buffer, and then, phosphorylated samples were resolved by SDS/PAGE and immunoblotting analysis with an anti‐phospho p65/RelA (S276) antibody (Rockland, Limerick, Japan).

### 2.9 Immunoblotting analysis

For usual immunoblotting analysis, cells were lysed with RIPA buffer without SDS [10 mm sodium phosphate (pH 7.2), 150 mm NaCl, 3 mm MgCl_2_, 2 mm EDTA, 1% NP‐40, 1% sodium deoxycholate, 0.2 U·mL^−1^ aprotinin, and phosphatase inhibitors] and briefly sonicated on ice. Then, debris were removed by sedimentation in a microcentrifuge at 16 400 ***g*** for 10 min, and cleared cell lysates were harvested and mixed with Laemmli sample buffer. Twenty‐five microgram of protein of whole cell lysates was loaded in each lane of an SDS‐polyacrylamide gel, and each protein was separated by the electrophoresis. Then, the separated proteins were transferred onto a polyvinylidene difluoride membrane (Merck Millipore). Proteins were visualized by immunoblotting analysis with the indicated antibodies and a chemical luminescence reagent, ECL (GE Healthcare). In some case such as the *in vitro* kinase assay, cells were lysed with NP‐40 lysis buffer as described above.

### 2.10 Quantitative RT‐PCR

Total RNA was extracted using TRIzol (Life Technologies, Waltham, MA, USA). To synthesize the single‐strand cDNA, 2 μg of total RNA was added into 20 μL reaction mixture including 100 units ReverTra Ace, 1 mm dNTPs, and 5 pmol oligo (dT)_20_ primer (TOYOBO, Osaka, Japan), and then, cDNA synthesis was performed for 60 min at 42 °C. The reaction was terminated by heating at 95 °C for 5 min and diluted with 80 μL TE buffer. One microlitre of synthesized cDNA was used for quantitative PCR in a 20 μL volume with the KAPA SYBR^®^ FAST qPCR Kit (KAPA Biosystems, Wilmington, MA, USA), and the reaction was analyzed by a LightCycler 96 (Roche diagnostics, Basel, Switzerland). The PCR primer sequences used are shown in Table S1.

### 2.11 Soft agar colony formation assay

For the colony formation assay, infected NIH‐3T3 cells were seeded onto soft agar at 1 × 10^4^ cells per 35‐mm‐diameter dish, and grown for 2–3 weeks. The visible colonies showing a diameter of 1.0 mm or more were counted using nih imagej software, freely provided from Dr. Wayne Rasband in NIH (https://imagej.nih.gov/ij/), and results were shown in the graph.

### 2.12 Analysis for human colorectal cancer samples

To detect K‐Ras mutations by quantitative PCR for the human colorectal tumor samples, and perform further experiments using them, we obtained informed consent forms from each patient. All experiments were undertaken with the understanding and written consent of each subject. The methodologies for experiments conformed to the standards set by the Declaration of Helsinki. The methodologies of current study were approved by the ethics committee in Jichi medical university.

### 2.13 Detection of K‐Ras mutations by quantitative PCR

From tumor tissues of each patient, genomic DNA was extracted using a NucleoSpin Tissue XS Genomic DNA Purification kit (Machery‐Nagel, Düren, Germany) in accordance with the manufacturer's instructions. The prepared genomic DNA was utilized for quantitative PCR analysis to detect K‐Ras mutations. Hydrolysis probes were designed in accordance with a previous report (van Eijk *et al.*, 2011). The mixtures were reacted in a LightCycler 96 (Roche Applied Science). For quantitative PCR, a VIC‐labeled hybridization probe was utilized to detect the wild‐type K‐Ras gene. On the other hand, FAM‐labeled hybridization probes were utilized to detect K‐Ras mutations such as G12S, G12R, G12C, G12D, G12A, G12V, and G13D. Detected K‐Ras mutations were colored purple (homozygous) or yellow (heterozygous). The sequences of primers and hydrolysis probes are shown in Table S2.

### 2.14 Immunohistochemical staining

To perform the immunohistochemical staining, tissue samples including tumor and normal tissues from 30 colorectal cancer (CRC) patients, and samples were fixed with formaldehyde. Among them, seven samples were selected for further analysis, because the tumor tissues in these samples harbored an oncogenic point mutation in exon 2 of the K‐Ras gene. The formalin‐fixed paraffin‐embedded sections were pretreated in a microwave oven for 15 min. First, H&E staining of each tumor tissue was performed to differentiate between tumor cell areas and normal tissues. Then, immunohistochemical staining was performed using anti‐p65/RelA or anti‐phospho p65/RelA (Ser‐276) as a primary antibody. As a secondary antibody, HRP‐conjugated anti‐rabbit IgG antibody (donkey) was used. Then, the sections were incubated with Envision (Agilent technology, Santa Clara, CA, USA) and stained with DAB (Wako, Osaka, Japan) for the visualization of signals.

### 2.15 Statistical analysis of data

All experiments were performed individually three times, and representative data shown. In graphs, error bars indicate standard deviation (SD, *n* = 3), and the results of calculations of independent *t*‐tests are shown.

## 3 Results

### 3.1 Oncogenic Ras mutants accelerate TNF‐α‐induced NF‐κB activation

**Figure 1:**
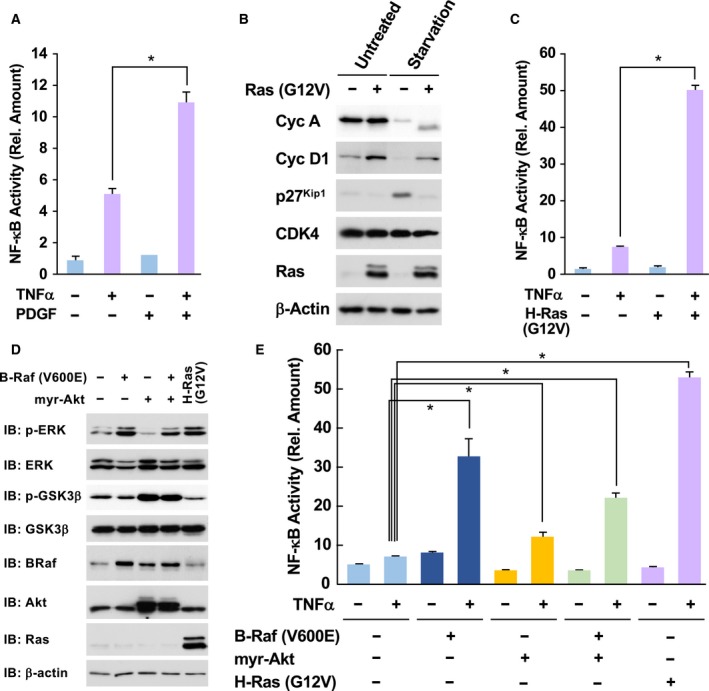
Oncogenic Ras mutants accelerate TNFα‐induced NF‐κB activation. (A) KF‐8 cells were cultured in DMEM without FBS for 24 h. Then, cells were stimulated with 10 μg·mL^−1^ TNFα with/without costimulation with PDGF (10 μg·mL^−1^) for 16 h. Cells were lysed, and the activity of expressed luciferase levels was measured as NF‐κB activation. (B) KF‐8 cells were infected with control retroviruses or retroviruses harboring H‐Ras (G12V). After puromycin selection, the expression of cell cycle‐related proteins, such as cyclin A (CycA), cyclin D1 (CycD1), Cdc2, CDK4, and H‐Ras, was detected by immunoblotting analysis. β‐actin was loading control. (C) Infected KF‐8 cells as shown in (B) were cultured in DMEM without FBS for 24 h. Cells were stimulated with 10 μg·mL^−1^ TNFα, and then, the activity of luciferase was analyzed. (D) KF‐8 cells were infected with control retroviruses or retroviruses harboring indicated cDNA. Then, downstream signals such as the phosphorylation of ERK and GSK3β were analyzed. (E) Using infected cells shown in (D), the luciferase assay was performed. In graphs, error bars indicate SD (*n* = 3), and the results of calculations of independent *t*‐tests are shown (*P < 0.005).

Previously, we established KF‐8 cells, a subline of NIH‐3T3 cells stably harboring κB‐responsive elements‐driven luciferase gene (Tago *et al.*, 2010). Using this cell line, we found that cell proliferative stimulation, such as by PDGF, augmented NF‐κB activation (Fig. 1A). This result suggested the presence of crosstalk between proliferative signals including oncogenic stimulation and the activation of NF‐κB. To test this, we transformed KF‐8 cells with a retrovirus harboring H‐Ras (G12V). As shown in Fig. 1B, the expression levels of cell cycle drivers such as cyclin A and cyclin D1 in KF‐8 cells were clearly silenced when serum in the medium was withdrawn (compare lane 1 and lane 3). In addition, the expression of p27^Kip1^, a marker protein for G_0_ phase, was enhanced by serum starvation. However, the enforced expression of H‐Ras (G12V) drastically enhanced the expression of cyclin A and cyclin D1, while the expression of p27^Kip1^ was markedly diminished.

Next, we investigated the effect of oncogenic signals on NF‐κB activation. Strikingly, when KF‐8 cells were stimulated with TNFα, luciferase activity was induced, and enforced expression of H‐Ras (G12V) caused the hyperacceleration of NF‐κB activation (Fig. 1C). However, H‐Ras (G12V) alone failed to activate NF‐κB. It was reported that murine fibroblasts can also be transformed by not only H‐Ras (G12V), but also mutated K‐Ras and c‐Myc (Keath *et al.*, 1984; Nakano *et al.*, 1984). A K‐Ras (G12V) mutant also accelerated TNFα‐induced NF‐κB activation; however, c‐Myc failed to affect NF‐κB activation (Fig. S1A). c‐Myc is involved in cell cycle progression and cellular transformation by Ras proto‐oncogenes, suggesting that c‐Myc‐provoked oncogenicity seems to be dispensable for the facilitation of the hyperactivation of NF‐κB. Current studies reported novel type of point mutation in K‐Ras gene, such as K117N and A146T (Yoshino *et al.*, 2015). These mutants also exhibited the activity to augment the TNFα‐induced NF‐κB activation (Fig. S1B). It is also well established that Ras stimulates multiple downstream molecules such as Raf and PI‐3 kinase, which activate downstream molecules including ERK and Akt (Boehm *et al.*, 2007; Calvisi *et al.*, 2011). To activate ERK or Akt in KF‐8 cells, we forcibly expressed B‐Raf (V600E) or N‐terminal myristoylated Akt (myr‐Akt), which are known as the constitutively active mutants of B‐Raf and Akt, respectively. As shown in Fig. 1D, enforced expression of B‐Raf (V600E) or myr‐Akt caused the activation of ERK or Akt, which were evaluated by the phosphorylation of ERK and GSK‐3β, respectively. Using these conditions, we found that the enforced expression of B‐Raf (V600E) strongly enhanced the TNFα‐induced activation of NF‐κB (Fig. 1E), whereas myr‐Akt only exhibited a slight enhancement of NF‐κB activation. These observations suggested that oncogenic Ras utilized the Raf‐dependent pathway but not Akt for the hyperactivation of NF‐κB.

### 3.2 Effect of oncogenic Ras mutant on TNF‐α‐induced signaling pathways

**Figure 2:**
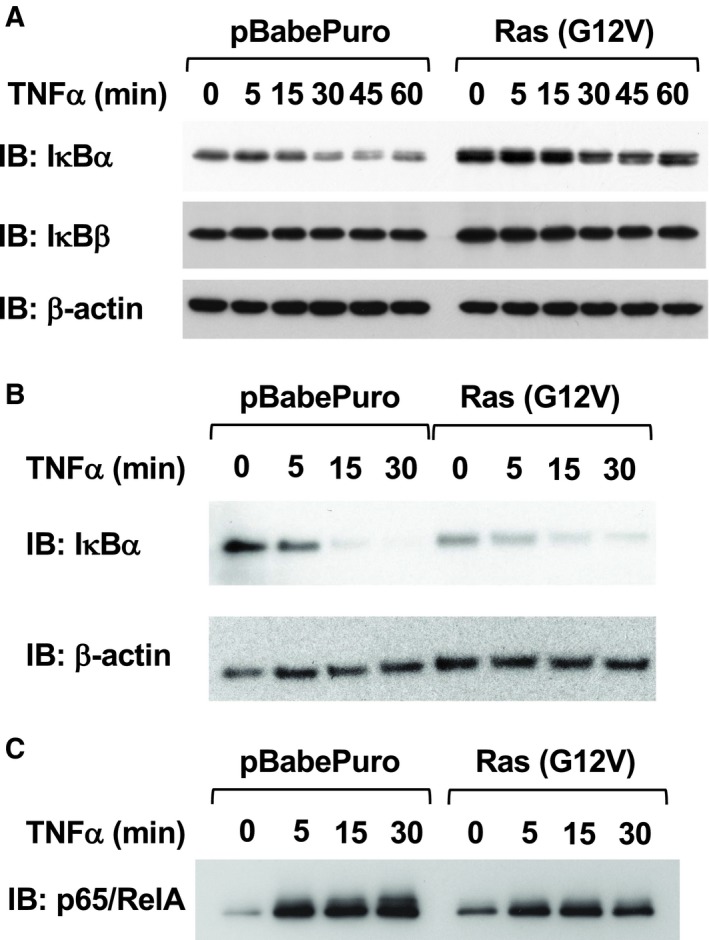
Effect of H‐Ras (G12V) on TNFα‐induced signaling pathways. (A) Parental NIH‐3T3 cells were infected with control retroviruses or retroviruses harboring H‐Ras (G12V). After puromycin selection, cells were stimulated with 10 μg·mL^−1^ TNFα for the indicated periods. Then, the cells were lysed, and the degradation of IκBα and IκBβ was analyzed by immunoblotting analysis. (B) Cells were treated with 25 μg·mL^−1^ CHX for 30 min, and then, the cells were stimulated with 10 μg·mL^−1^ TNFα for the indicated periods. Cell lysates were prepared, and the degradation of IκBα was analyzed. (C) From the infected NIH‐3T3 cells shown in (A), nuclear extracts were prepared. The nuclear extracts were incubated with a biotin‐labeled DNA probe harboring κB‐responsive elements, and then, DNA·protein complexes were captured using streptavidin‐conjugated agarose. The captured proteins were eluted with sample buffer. Using these samples, the nuclear localization and DNA binding activity of NF‐κB were evaluated by immunoblotting analysis for p65/RelA.

Next, we investigated the effect of H‐Ras (G12V) on TNF‐α‐induced signal transduction pathways for NF‐κB activation. As shown in Fig. 2A, we analyzed the effect of H‐Ras (G12V) on the degradation of IκBα and IκBβcaused by TNFα. In Ras‐provoked transformed NIH‐3T3 cells, expression of IκBα protein was slightly enhanced, and the TNFα‐induced degradation of IκBα seemed to be attenuated. In addition, the degradation of another negative regulator, IκBβ, was not observed in NIH‐3T3 cells stimulated with TNFα. As reported in previous studies, the IκBα gene was identified as a target gene of NF‐κB (Sun *et al.*, 1993); therefore, it was suggested that the enhancement of IκBα expression caused the apparent attenuation of degradation of IκBα in Ras‐transformed cells. Therefore, we next tested the effect of Ras‐provoked transformation on the degradation of IκBα in the cells treated with cycloheximide (CHX), as an inhibitor of protein synthesis. In this experimental condition, we only observed the degradation of IκBα; however, we still could not observe its acceleration by oncogenic Ras (Fig. 2B). Next, to evaluate the nuclear localization and DNA binding activity of NF‐κB, we performed a DNA pull‐down assay. The nuclear extracts were mixed with biotinylated DNA probe including κB‐responsive elements, and complexes formed, including active p65/RelA, were captured by affinity resin conjugated with streptavidin. Then, p65/RelA bound to the DNA probe was detected by immunoblotting. As shown in Fig. 2C, the nuclear translocation of NF‐κB was not accelerated by Ras‐provoked cellular transformation.

### 3.3 Ras (G12V)‐induced hyperactivation of NF‐κB requires cell cycle progression

**Figure 3:**
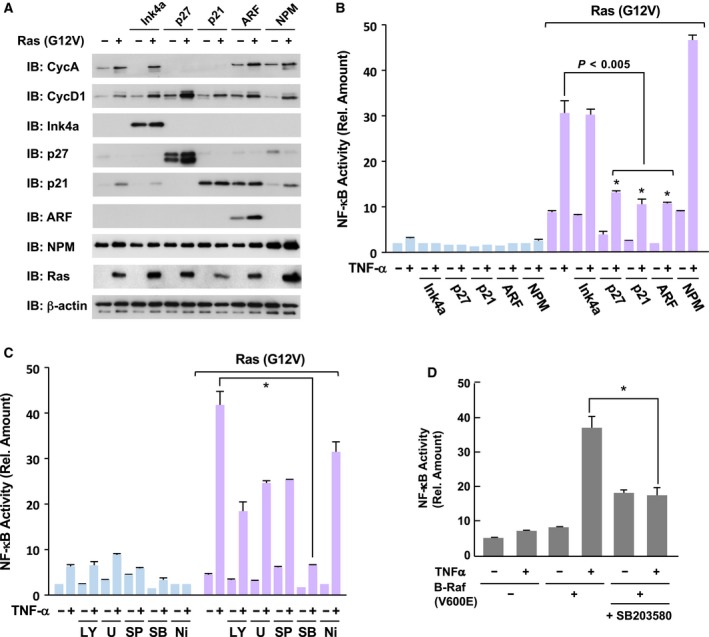
Oncogenic NF‐κB activation requires cell cycle progression and p38. (A) KF‐8 cells were infected with control retroviruses or retroviruses harboring indicated cDNA. After puromycin selection, cell lysates were prepared and analyzed by immunoblotting analysis using the indicated antibodies. (B) Infected KF‐8 cells were cultured in DMEM without FBS for 24 h and then stimulated with 10 μg·mL^−1^ TNFα for 16 h. Then, using these cells, the activity of luciferase was analyzed to evaluate NF‐κB activation. (C) KF‐8 cells infected with control retroviruses or retroviruses harboring H‐Ras (G12V) were treated with the indicated inhibitors for 30 min. Then, the cells were stimulated with 10 μg·mL^−1^ TNFα for 16 h and utilized for the luciferase assays to evaluate NF‐κB activation. Inhibitors LY294002 (LY, 20 μm), U0126 (U, 20 μm), SP600125 (SP, 50 μm), SB203580 (SB, 10 μm), and VAS2870 (Ni, 10 μm) were used. (D) KF‐8 cells were infected with control retroviruses or retroviruses harboring B‐Raf (V600E). By performing luciferase assays, the effect of SB203580 (10 μm) on B‐Raf (V600E)‐provoked hyperactivation of NF‐κB was analyzed. In graphs, error bars indicate SD (*n* = 3), and the results of calculations of independent *t*‐tests are shown (*P < 0.005).

Previously, it was reported that the cyclin‐dependent kinase (CDK) inhibitor p16^Ink4a^ but not p27^Kip1^ suppressed TNFα‐induced NF‐κB activation in HEK293 and HeLa cells (Wolff and Naumann, 1999). To clarify the functional involvement of cell cycle progression induced by oncogenic Ras in the hyperactivation of NF‐κB, we next investigated the effects of CDK inhibitors on the oncogenic activation of NF‐κB in Ras mutant‐provoked transformed cells. Using the retroviral technique, we forcibly expressed p16^Ink4a^, p27^Kip1^, p21^Cip1^, and p19^ARF^ tumor suppressors. As shown in Fig. 3A, Ras (G12V) resulted in enhanced expression of cell cycle drivers such as cyclin A and cyclin D1. Enforced expression of p27^Kip1^ and p21^Cip1^ suppressed the expression of cyclin A. In addition, p19^ARF^ induced the expression of p21, which was probably mediated by the activation of p53. In these conditions, we evaluated the activation of NF‐κB stimulated by Ras (G12V) and TNFα. Unexpectedly, we observed that the enforced expression of p16^Ink4a^ could not inhibit the hyperactivation of NF‐κB (Fig. 3B). On the other hand, p27^Kip1^ and p21^Cip1^ drastically suppressed the hyperactivation of NF‐κB. Furthermore, p19^ARF^ also exhibited inhibitory effects on the hyperactivation of NF‐κB. Nucleophosmin (NPM) is a marker protein in the nucleolus, and some studies reported that NPM antagonizes p19^ARF^ (Itahana *et al.*, 2003). Of note, exogenous expression of NPM effectively enhanced the hyperactivation of NF‐κB by co‐stimulation with TNFα and Ras (G12V). These observations strongly suggested that the oncogenic hyperactivation of NF‐κB requires cell cycle progression.

### 3.4 Oncogenic acceleration of NF‐κB activation requires p38 MAP kinase

Next, we investigated the functional involvement of MAP kinases and PI‐3 kinase in the hyperactivation of NF‐κB provoked by oncogenic Ras mutant. As shown in Fig. 3C, several protein kinase inhibitors, such as LY294002 (20 μm), U0126 (20 μm), and SP600125 (50 μm), slightly suppressed the hyperactivation of NF‐κB, suggesting the partial involvement of the PI‐3 kinase, ERK, and JNK pathways. Mitsushita *et al. *(2004) also reported the requirement of NOX1, an NADPH oxidase for Ras‐provoked transformation. However, VAS2870 (10 μm), an inhibitor for NOX1, exhibited slight inhibitory effect on NF‐κB. Notably, treatment with 10 μm of SB203580, an inhibitor of p38 MAP kinase, exhibited drastic inhibitory effects on the oncogenic hyperactivation of NF‐κB. These observations suggested the functional involvement of p38 MAP kinase in the H‐Ras (G12V)‐provoked oncogenic hyperactivation of NF‐κB. As shown in Fig. 1E, an oncogenic downstream effector of Ras, B‐Raf (V600E), also possessed the ability to cause the hyperactivation of NF‐κB. Strikingly, 10 μm of SB203580 effectively suppressed the hyperactivation of NF‐κB induced by co‐stimulation with B‐Raf (V600E) and TNFα (Fig. 3D). It was established that p38 MAP kinase is functionally involved in oncogenic Ras‐ or growth stimulation‐induced premature senescence in human fibroblasts (Debacq‐Chainiaux *et al.*, 2010; Yaswen and Campisi, 2007). In addition, our experimental results shown in Fig. 3A,B clearly demonstrated the requirement of cell cycle progression for the hyperactivation of NF‐κB, suggesting that Ras‐provoked cell cycle progression and p38 activation cooperatively facilitate the activation of NF‐κB.

### 3.5 Oncogenic Ras accelerates the transcriptional activation of NF‐κB

**Figure 4:**
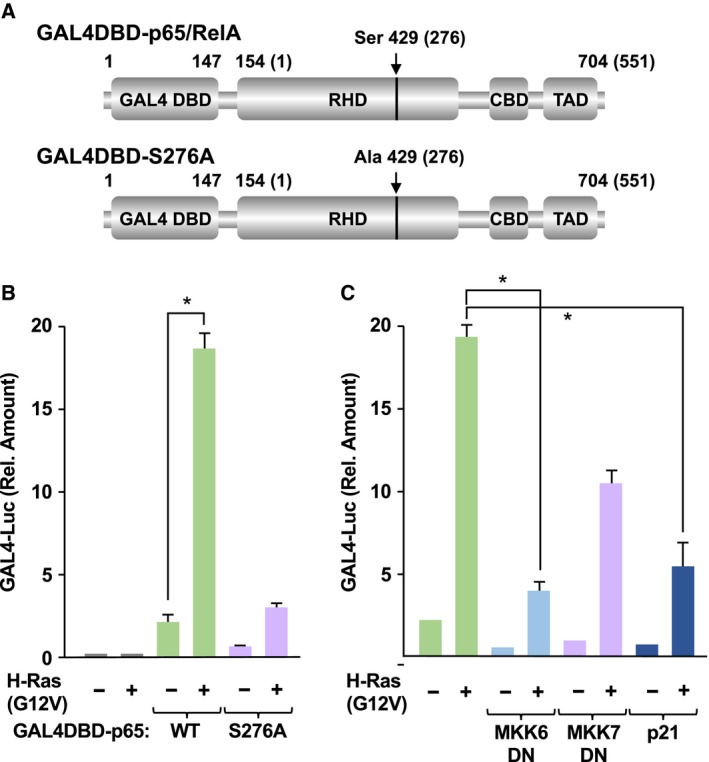
Oncogenic Ras mutants accelerate the transcriptional activation of p65. (A) Schemes of GAL4DBD‐p65/RelA and GAL4DBD‐p65/RelA (S276A) are shown. RHD, CBD, and TAD indicate the RHD, CBP/p300‐binding domain, and transcriptional activating domain, respectively. Numbers in parentheses indicate the original position of amino acids in p65/RelA. (B) NIH‐3T3 cells were transfected with pFR‐luciferase vector with the indicated combination of plasmids harboring GAL4DBD‐p65/RelA, GAL4DBD‐p65/RelA (S276A), H‐Ras (G12V). The cells were cultured in FBS‐free DMEM for 24 h, then stimulated with TNFα for 16 h. Cells were harvested, and their luciferase activity was measured. (C) Using the same experimental conditions with (B), the effects of dominant negative mutants of MKK6 and MKK7 and CDK inhibitor p21^Cip1^ on H‐Ras (G12V)‐provoked transcriptional activation of p65/RelA were analyzed. In graphs, error bars indicate SD (*n* = 3), and the results of calculations of independent *t*‐tests are shown (*P < 0.005).

As shown in Fig. 2, oncogenic Ras mutants did not accelerate the degradation of IκBα and nuclear translocation of the p65/RelA subunit. Finco *et al. *(1997) reported that oncogenic H‐Ras mutant activates NF‐κB transcriptional activity. In addition, to investigate the transcriptional activation of NF‐κB, we previously constructed an expression plasmid for fusion protein of p65/RelA, harboring the DNA binding domain of GAL4 transcription factor on the N terminus of p65/RelA, named GAL4DBD‐p65/RelA (Fig. 4A; Tago *et al.*, 2010). Therefore, we transfected the expression vectors for GAL4DBD‐p65/RelA with a reporter plasmid harboring a GAL4‐responsive element (pFR‐luciferase) into NIH‐3T3 cells and evaluated the expression of luciferase as the transcriptional activity of p65/RelA. As shown in Fig. 4B, H‐Ras (G12V) strongly augmented the transcriptional activity of GAL4DBD‐p65/RelA, suggesting that the Ras‐provoked oncogenic signal accelerates the transcriptional activation of NF‐κB. In the case of p65/RelA, phosphorylation of the serine residue at 276 (S276) accelerates the interaction between the NF‐κB complex and p300/CBP (Tago *et al.*, 2010; Zhong *et al.*, 1998). The interaction between the NF‐κB complex and p300/CBP is well known to be required for the transcriptional activation of NF‐κB. Therefore, we investigated the effect of oncogenic Ras on the transcription activity of a GAL4DBD‐p65/RelA (S276A) mutant, in which serine at 276 in p65/RelA was substituted with alanine. H‐Ras (G12V) failed to accelerate the transcriptional activity of GAL4DBD‐p65/RelA (S276A). Furthermore, the transcriptional activity of GAL4DBD‐p65/RelA enhanced by H‐Ras (G12V) was drastically diminished by the expression of the dominant negative mutant of MKK6 (MKK6DN), which functions as an upstream kinase of p38 MAP kinase (Raingeaud *et al.*, 1996) and p21^Cip1^ CDK inhibitor (Fig. 4C). On the other hand, the dominant negative mutant of MKK7 (MKK7DN), which functions as an upstream kinase of JNK, moderately inhibited the oncogenic Ras mutant‐induced activation. These observations suggest that the oncogenic Ras‐enhanced transcriptional activation of NF‐κB seems to be mediated by the phosphorylation of Ser‐276 in the p65/RelA subunit, and this phosphorylation requires the activation of p38 MAP kinase and cell cycle progression.

### 3.6 Ras‐p38‐MSK1/2 signaling axis contributes to the hyperactivation of NF‐κB

**Figure 5:**
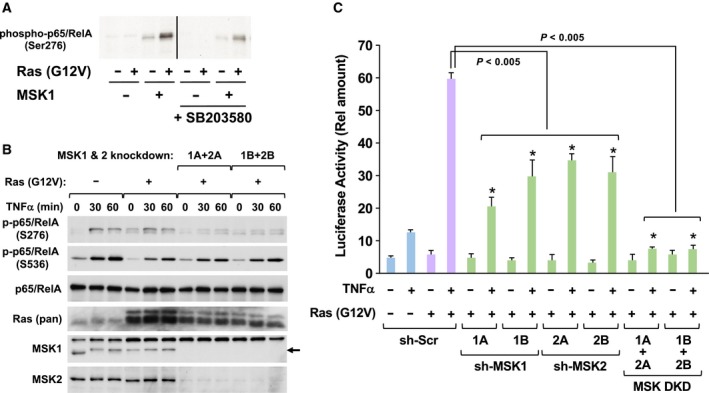
MSK1/2 contributes to the oncogenic transcriptional activation of NF‐κB. (A) HEK293T cells were transfected with the indicated combinations of plasmids harboring FLAG‐MSK1 and H‐Ras (G12V). The cells were cultured in FBS‐free DMEM for 24 h, and then, the cells were lysed with NP‐40 lysis buffer. FLAG‐MSK1 was purified by immunoprecipitation with M2‐agarose, then incubated with 3 μg GST‐p65/RelA and 100 μm ATP at 30 °C for 30 min. The phosphorylation of p65/RelA at Ser276 was detected by immunoblotting analysis using an antibody against phosphorylated p65/RelA (S276). (B) NIH‐3T3 cells infected with indicated retroviruses were stimulated with 10 ng·mL^−1^ TNFα for indicated periods, and then, their cell lysates were prepared as described in Materials and Methods. The phosphorylation of p65/RelA at Ser‐276 and Ser‐536 was detected by immunoblot analysis using indicated antibodies, respectively. The expressions of p65/RelA and H‐Ras were also detected by immunoblot analysis using indicated antibodies. (C) KF‐8 cells were infected with the indicated combinations of retroviruses harboring H‐Ras (G12V), shRNA, sh‐MSK1, or sh‐MSK2. Then, using these infected cells, luciferase assays were performed. In graph, error bars indicate SD (*n* = 3), and the results of calculations of independent *t*‐tests are shown (*P < 0.005).

In previous studies, cAMP‐dependent protein kinase A (PKA) and MSK1, a downstream protein kinase of p38 MAP kinase, were reported to be protein kinases for the phosphorylation of p65/RelA at Ser‐276 (Joo and Jetten, 2008; Zhong *et al.*, 1997). However, we observed that the enforced expression of the catalytic subunit of PKA (PKAc) strongly inhibited TNFα‐induced NF‐κB activation. Therefore, we concluded that PKA is not involved in TNFα‐induced NF‐κB activation (Fig. S2A). We next investigated whether MSK1 is involved in the transcriptional activation of NF‐κB mediated by the phosphorylation of p65/RelA at Ser‐276. Using a transient transfection technique, we forcibly expressed MSK1 with or without H‐Ras (G12V) in HEK293T cells (Fig. S2B), and then, MSK1 was purified by immunoprecipitation and its kinase activity was evaluated using an *in vitro* kinase assay with recombinant protein of GST‐fused p65/RelA as the substrate. The phosphorylation of GST‐p65/RelA was detected by immunoblotting using an antiphosphorylated p65/RelA at Ser‐276 antibody. As shown in Fig. 5A, we observed that MSK1 exhibited weak kinase activity against p65/RelA, and its kinase activity was strongly enhanced by the co‐expression of H‐Ras (G12V). Furthermore, the activation of MSK1 by Ras (G12V) was effectively suppressed by a p38 inhibitor, SB203580, suggesting that MSK1 seems to specifically phosphorylate p65/RelA at Ser‐276 for the oncogenic hyperactivation of NF‐κB. We next analyzed whether the phosphorylation of endogenous p65/RelA at Ser‐276 is suppressed by the knockdown of MSK1/2 (Fig. 5B). We constructed retroviral vectors including shRNA sequences against MSK1 and its related kinase MSK2, and these shRNA successfully silenced the expression of MSK1 and MSK2 (Fig. S2C). Strikingly, Ras (G12V) alone induced the phosphorylation of p65/RelA at Ser‐276 without stimulation with TNFα (see lane 4). The stimulation with TNFα caused the Ser‐276 phosphorylation, and this phosphorylation was slightly augmented by oncogenic Ras mutant. Strikingly, the Ser‐276 phosphorylation of p65/RelA was drastically diminished by the knockdown of MSK1 and MSK2. The knockdown of MSK1/2 did not affect the transforming activity provoked by Ras (G12V) (Fig. S3), suggesting that MSK1/2‐p65/RelA signaling axis does not contribute to cancer initiation. In addition, we tested whether silencing MSK1 expression affected the activation of NF‐κB. Using retroviruses including these shRNA, we tested their effect on the activation of NF‐κB stimulated by TNFα and Ras (G12V). As shown in Fig. 5C, shRNA against MSK1 or MSK2 effectively inhibited the oncogenic hyperactivation of NF‐κB. Furthermore, combined expression of shRNA against MSK1 and MSK2 completely suppressed the oncogenic hyperactivation of NF‐κB. These observations strongly suggested that the Ras‐p38‐MSK1/2 signaling axis contributes to the transcriptional activation of NF‐κB in murine fibroblasts.

### 3.7 Oncogenic signal provoked by K‐Ras mutants is required for the NF‐κB activation in cancer cell line

**Figure 6:**
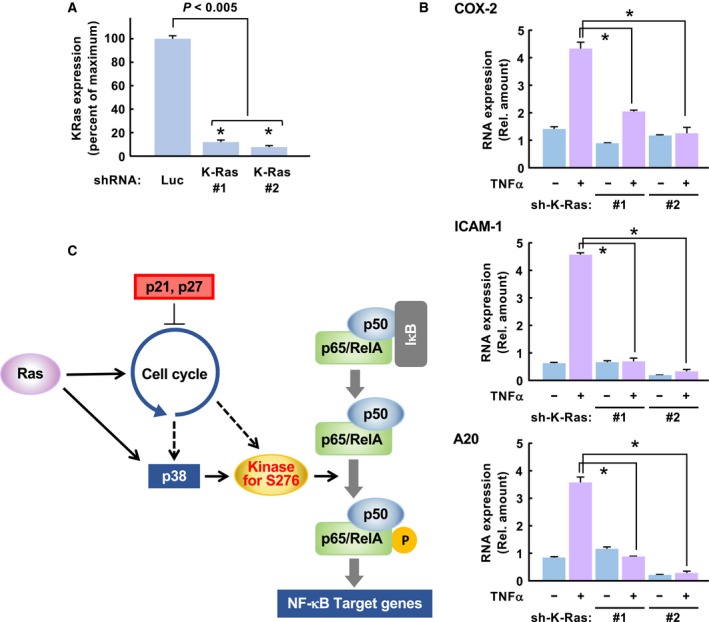
Oncogenic K‐Ras signal is also required for NF‐κB activation in a human cell line. (A) Using retroviruses including shRNA against K‐Ras, the expression of K‐Ras mRNA was silenced in A549, a human lung cancer cell line. The amount of K‐Ras mRNA was evaluated using quantitative RT‐PCR (qPCR). (B) A549 cells were infected with retroviruses harboring sh‐luciferase (sh‐Luc) or K‐Ras. The cells were stimulated with 10 μg·mL^−1^ TNFα for 3 h, then harvested for the extraction of total RNA. Using the total RNA, qPCR was performed to evaluate the effect of knockdown of oncogenic K‐Ras on the TNFα‐induced expression of NF‐κB target genes such as COX‐2, ICAM1, and A20. In graphs, error bars indicate SD (*n* = 3), and the results of calculations of independent *t*‐tests are shown (*P < 0.005). (C) Our observations summarize the mechanism how oncogenic Ras causes hyperactivation of NF‐κB. In the case of murine fibroblasts, Kinase for S276 is MSK1 and MSK2.

In addition, we tested the requirement for an oncogenic Ras signal for NF‐κB activation in human cancer cells. We infected retroviruses harboring shRNA against K‐Ras to knockdown K‐Ras protein in A549 cells, a lung cancer‐derived cell line, which harbors a homozygous G12S mutation in the K‐Ras gene (Mitsudomi *et al.*, 1991) (Fig. 6A). Then, the expression of NF‐κB target genes, such as COX‐2, A20, and intercellular adhesion molecule‐1 (ICAM‐1) induced by stimulation with/without TNFα, was analyzed by quantitative RT‐PCR. Strikingly, the knockdown of K‐Ras protein strongly diminished the induction of expression of COX‐2, A20, and ICAM‐1 by the stimulation with TNFα (Fig. 6B). These observations strongly support that Ras‐provoked oncogenic signal is required for the activation of NF‐κB in cancer cells. Taking all our observations, we summarized the mechanism how oncogenic Ras mutant causes the hyperactivation of NF‐κB (Fig. 6C).

### 3.8 Presence of MSK1/2‐independent machinery for NF‐κB activation in human cancer cell line

**Figure 7:**
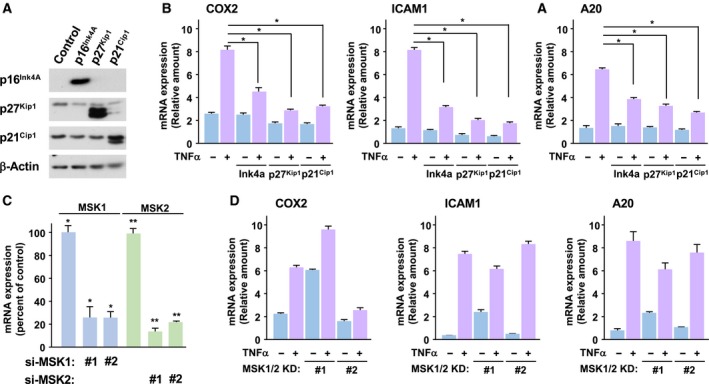
Presence of MSK1/2‐independent machinery for NF‐κB activation in human cancer cell line. (A) A549 cells were infected with the retroviruses including cDNA of Ink4a, p27^Kip1^, and p21^Cip1^. The expressions of these proteins were observed by immunoblot analysis using indicated antibodies. (B) The infected A549 cells were stimulated with 10 ng·mL^−1^ TNFα for 3 h, and then, total RNA were extracted. Using these total RNA, the expressions of COX‐2, ICAM1, and A20 were analyzed by quantitative RT‐PCR. (C) A549 cells were transfected with siRNA against both human MSK1 (1A or 1B) and MSK2 (2A or 2B). And then, the cells were stimulated with TNFα as described above, and then, total RNA were extracted. The knockdown of MSK1 (blue bar) and MSK2 (green bar) was evaluated by the quantitative RT‐PCR. (D) The expressions of COX‐2, ICAM1, and A20 in the cells shown in (C) were analyzed by the quantitative RT‐PCR. In graphs, error bars indicate SD (*n* = 3), and the results of calculations of independent *t*‐tests are shown (*P < 0.005).

Next, we tested whether the oncogenic K‐Ras mutant utilized similar signaling pathway for the activation of NF‐κB with the case of NIH‐3T3 cells. As shown in Fig. 7A,B, TNFα‐induced activation of NF‐κB was significantly inhibited by the expression of Ink4a, p27^Kip1^, and p21^Cip1^, suggesting the requirement of cell cycle progression for the NF‐κB activation in A549 cells. In addition, we also tested the effect of knockdown of MSK1/2 by siRNA on the NF‐κB activation. However, it was unexpected that silencing the expression of MSK1/2 caused no alteration on the TNFα‐induced expression of NF‐κB target genes (Fig. 7C,D). In the case of SW620 cells, a CRC‐derived cell line harboring G12V mutation in K‐Ras gene, it was also observed that the TNFα‐induced expressions of COX‐2, A20, and ICAM1 were not affected by the knockdown of MSK1/2 (Fig. S4). We do not have suitable explanation for the difference of the experimental results for the effect of MSK1/2 knockdown on NF‐κB activation from the case of NIH‐3T3 cells. However, our observations clearly showed the presence of MSK1/2‐independent machinery for the NF‐κB activation in human cancer cell lines.

### 3.9 Hyperactivation of NF‐κB in colorectal cancer harboring K‐Ras mutation

**Figure 8:**
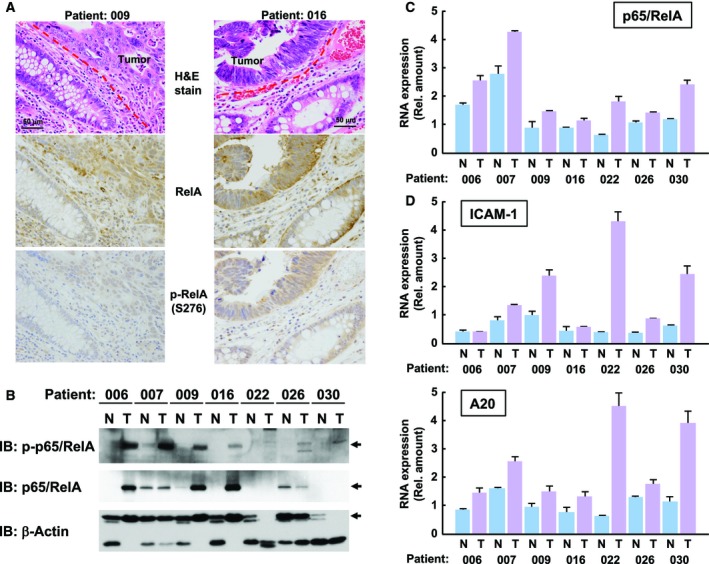
Hyperactivation of NF‐κB by K‐Ras mutations in human cancer tissues. (A) Utilizing paired samples of tumor and normal tissues from K‐Ras (+) patients, immunohistochemical analyses were performed to detect the total p65/RelA and phosphorylated p65/RelA (Ser276). H&E staining is also shown. In the photograph, 50 μm scale bar was shown. (B) Extracts from tumor tissues and normal mucous membrane tissues were analyzed by immunoblotting analysis with anti‐phospho‐p65/RelA (S276), antitotal p65/RelA, and anti‐β‐actin antibodies. In the results, tumor and normal samples are shown labeled as T and N, respectively. (C) Using total RNA extracted from tumor tissues and normal mucous membrane tissues shown in (B), the mRNA expression levels of p65/RelA were analyzed by qPCR. In the graph, error bars = SD (*n* = 3). (D) Using same cDNA with (C), quantitative RT‐PCR was performed to evaluate the mRNA expression of ICAM‐1 and A20 in tumor and normal tissues, respectively. In the graph, error bars = SD (*n* = 3).

In CRC patients, 40% of patients’ samples harbored a point mutation of K‐Ras gene (Bos, 1989; Naser *et al.*, 2014). Finally, we investigated whether oncogenic mutation of the K‐Ras gene in CRC causes phosphorylation of the p65/RelA subunit at Ser‐276. To find CRC samples harboring an oncogenic mutation of K‐Ras gene, we employed an assay system to screen for point mutations of the K‐Ras gene at codon 12 and 13 using quantitative PCR, as reported previously (van Eijk *et al.*, 2011). Among 30 CRC patients’ samples, we found that seven patients harbored an oncogenic mutation of the K‐Ras gene, which were termed K‐Ras (+) patients (Table S1 and Fig. S5). Utilizing paired samples of tumor and normal tissues from these K‐Ras (+) patients, we performed immunohistochemical analyses to test for the presence of phosphorylated p65/RelA at Ser‐276. Of note, higher phosphorylation of p65/RelA at Ser‐276 was observed only in tumor samples/areas rather than in normal colorectal tissues in several K‐Ras (+) CRC patients, including patients 006, 009, 016, 026, and 030 (Fig. 8A and Fig. S6). To confirm enhancement of the phosphorylation of p65/RelA at Ser‐276, we performed immunoblotting analysis. As shown in Fig. 8B, the phosphorylation of p65/RelA at Ser‐276 was observed only in tumor samples but not in normal mucous membrane tissues. Unexpectedly, we observed that the total protein amounts of p65/RelA were also enhanced in tumor samples/areas, whereas the protein was undetectable in several cases of normal mucous membrane. However, the mRNA expression levels of p65/RelA were similar between cancer and normal tissues, suggesting the differences in protein expression of p65/RelA between cancer and normal tissues seemed to be due to the protein stability or translational efficiency of p65/RelA (Fig. 8C). In the immunoblotting analysis, we analyzed β‐actin as a loading control evaluating the protein amount and quality in each sample, and we detected truncated/degraded β‐actin in several samples, such as patients 022, 026, and 030. Finally, we analyzed the mRNA expression levels of NF‐κB target genes, COX‐2, A20, ICAM‐1, and VEGF. As shown in Fig. 8D and Fig. S7, the expression of COX‐2 was enhanced in cancer tissues of patients 022, 026, and 030 but not in other patients. On the other hand, the mRNA expression levels of A20 and ICAM‐1 were enhanced in many cancer tumor samples, and especially, enhancement of A20 expression was observed in all tumor samples. These data strongly supported our model shown in Fig. 6C.

## 4 Discussion

Conserved residues in the trans‐activation domain of the p65/RelA subunit, which can be post‐translationally modified by phosphorylation, acetylation, and conjugation with ubiquitin family proteins, and these modifications regulated divergent NF‐κB functions in response to different cellular stimulations (Perkins, 2006). Among these post‐translational modifications of the p65/RelA subunit, the importance of p65/RelA phosphorylation has been reported by numerous researchers. Duran *et al. *(2003) reported that phosphorylation of p65/RelA at Ser‐311 by PKCζ disrupts the interaction with Euchromatic histone‐lysine *N*‐methyltransferase 1 (EHMT1), which catalyzes monomethylation of p65/RelA at Lys‐310 and suppresses the expression of NF‐κB target genes. Additionally, several studies reported the importance of the phosphorylation of p65/RelA at Ser‐536 for the nuclear translocation or transcriptional activation of NF‐κB (Sakurai *et al.*, 1999; Sasaki *et al.*, 2005), which are demonstrated by the effect of a p65 mutant in which Ser‐536 was substituted by alanine (S536A). However, Pradère *et al. *(2016) utilized knock‐in mice and demonstrated the activity of NF‐κB was negatively regulated by the phosphorylation of p65/RelA at Ser‐536.

The p65/RelA subunit is known to be phosphorylated at several serine/threonine residues in order to exhibit its transcriptional activity. The phosphorylation of p65/RelA at Ser‐276 was reported to be a trigger for the interaction between p65/RelA and transcriptional coactivator p300 and CBP (Tago *et al.*, 2010; Zhong *et al.*, 1998). The recruitment of p300/CBP onto NF‐κB complexes results in the acetylation of p65/RelA at Lys‐310, which causes the blockage of IκB‐induced export of NF‐κB from the nucleus (Chen *et al.*, 2001). Therefore, the protein kinases phosphorylating p65/RelA at Ser‐276 should be critical for the activation of NF‐κB. PKAc and MSK1 were reported to harbor kinase activity toward p65/RelA at Ser‐276 (Joo and Jetten, 2008; Zhong *et al.*, 1997). In many cases, activation of PKA was triggered by the elevation of cytosolic cAMP and followed conformational alteration of PKA complexes (Smith *et al.*, 2017). However, Zhong et al. reported that PKA could be activated by stimulation with TNFα in a cAMP‐independent manner (Zhong *et al.*, 1997). In our current results, MSK1 exhibited the ability to phosphorylate p65/RelA at Ser‐276. Strikingly, the kinase activity of MSK1 was drastically accelerated by oncogenic Ras, and this activation was diminished by p38 inhibitor. On the other hand, enforced expression of PKAc negatively regulated NF‐κB activation (Fig. S2). Furthermore, the combined knockdown of MSK1 and MSK2 completely suppressed the hyperactivation of NF‐κB, suggesting that oncogenic Ras utilizes the p38‐MSK1/2 signaling axis for the oncogenic hyperactivation of NF‐κB (Fig. 5C). However, it is still unclear how p38 MAP kinase is activated by oncogenic Ras, in spite of several studies reporting the functional involvement of p38 in Ras signal (Debacq‐Chainiaux *et al.*, 2010; Yaswen and Campisi, 2007). On the other hand, Ras signal seems to utilize MSK1/2‐independent signaling pathway for the activation of NF‐κB in A549 and SW620 cells (Fig. 8 and Fig. S4). Now, we have no idea these differences in the results were due to the species of cell lines, cell types, or anything else. However, it must be important to solve these problems to clarify the mechanism how Ras signal accelerates transcriptional activation of NF‐κB, and these studies would provide clues for the development of novel anticancer drugs.

As shown in Fig. 8A,B, the phosphorylation of p65/RelA at Ser276 was strongly enhanced in tumor tissues. However, the protein amount of total p65/RelA was unexpectedly upregulated in tumor tissues. This was not correlated with the expression of p65/RelA mRNA, suggesting that the enhancement of the protein expression of p65/RelA seems to be due to protein stabilization or translational acceleration and not mRNA transcription. Our observation suggested the presence of unknown machinery enhancing the protein amount of p65/RelA in tumor samples. We also showed that the expression levels of NF‐κB target genes were enhanced in several tumor tissues. However, the pattern of enhancement of NF‐κB activity was different in each target gene (Fig. 8C). Currently, we do not have a clear explanation for this discrepancy; however, it is well understood that gene expression in malignant tumors is also regulated by epigenetic machinery such as modification of DNA and histones (Esteller, 2007). In addition, functional crosstalk with other transcription factors might cause an alteration in the expression pattern of NF‐κB target genes in tumors, such as the case of HIF1α for the activation of the VEGF promoter (Giatromanolaki *et al.*, 2001). In future investigations, it will be necessary to clarify the relationship between the activation of NF‐κB in tumors and their prognosis, and the MSK1/2‐caused phosphorylation of p65/RelA at Ser‐276 would be an important clue for clarifying these problems.

In addition, we would like to discuss about possible involvement of noncanonical pathway of NF‐κB in our system. Vreka *et al. *(2018) reported that IKKα‐RelB signal was required for development and progression of lung cancer harboring K‐Ras mutation. On the other hand, Song *et al. *(2018) reported that IKKα functions as tumor suppressor against the development of K‐Ras‐initiated lung cancer. The differences between these two reports suggest the presence of diversified NF‐κB signaling pathway in Ras‐provoked carcinogenesis. In the current study, we did not test the possibility that IKKα‐RelB signal is involved in the Ras‐induced activation of NF‐κB. In future, it will be required for understanding details of Ras‐induced tumor development and progression to study the possible involvement of noncanonical NF‐κB signaling pathways.

## 5 Conclusions

In the current study, we focused on the molecular mechanism how oncogenic Ras mutants augments the transcriptional activity of NF‐κB. Oncogenic hyper‐activation of NF‐κB requires cell cycle progression. Oncogenic Ras‐provoked hyperactivation of NF‐κB is triggered by p38‐MSK1/2 signaling axis, which enhances the transcriptional activation of NF‐κB via the phosphorylation of RelA at Ser‐276. The phosphorylation of RelA at Ser‐276 is enhanced in CRC tissues.

## Conflict of interest

The authors declare no conflict of interest.

## Author contributions

KT and MF‐T performed all of the experiments except quantitative RT‐PCR and immunohistochemical staining. SO prepared retroviruses. HS contributed to purify the recombinant protein of GST‐p65/RelA for *in vitro* kinase assay of MSK1 and developed its assay system. CA‐O contributed to the analysis of qPCR. HK and AT performed immunohistochemical staining of tumor samples from colorectal cancer patients and analyzed the results. HH contributed to preparation of tumor samples from colorectal cancer patients by surgery. CA‐O and JY contributed to plasmids construction. KT, JM, and KY analyzed data. KT, MF‐T, and KY wrote the manuscript.

## Supporting information


**Fig. S1.** (A) KF‐8 cells were infected with control retroviruses or retroviruses harboring K‐Ras (G12V) or c‐Myc. After puromycin selection, cells were stimulated with 10 μg/ml TNFa for indicated periods. Then, cells were lysed, and the luciferase assay was performed. (B) Using KF‐8 cells infected with control retroviruses or retroviruses harboring K‐Ras mutants including G12V, K117N and A146T, the luciferase assay was performed as shown in (A). In the graph, error bars = SD (n = 3, *P < 0.005).
**Fig. S2.** (A) HEK293T cells were transfected with a luciferase vector including κB‐responsive element with/without FLAG‐PKAc, a catalytic subunit of PKA. Then, using the transfected cells, luciferase assays were performed. In graph, error bars indicate standard deviation (S.D., n = 3), and the results of calculations of independent t‐tests are shown (*P < 0.005). (B) The expression of FLAG‐MSK1 and H‐Ras (G12V) in cells analyzed for *in vitro *kinase assay (Figure 5A) was analyzed by immunoblotting analysis using antibodies against FLAG‐tag (M2) and H‐Ras. (C) KF‐8 cells were infected with retroviruses harboring scrambled short‐hairpin RNA, sh‐MSK1 (1A or 1B) or sh‐MSK2 (2A or 2B). After puromycin selection, cells were lysed, and immunoblotting analysis was performed to evaluate the knockdown efficiency of MSK1 and MSK2. β‐actin was used as a loading control.
**Fig. S3.** (A) NIH‐3T3 cells were infected with indicated retroviruses as described in Figure 5B. After puromycin selection, cells (1 × 104 cells) were seeded on soft agar media in 35 mm dishes. After 3 weeks, the number of colonies were counted and shown in graph as shown in (B). In the graph, error bars = SD (n = 3, *P < 0.005). In the photograph, 500 μm scale bar was shown.
**Fig. S4.** SW620, a colorectal cancer‐derived cell line was transfected with siRNAs against both MSK1 and MSK2.
**Fig. S5.** Genomic DNA was extracted from tumor tissues of 30 colorectal cancer patients (designated as T001 to T030).
**Fig. S6.** Utilizing paired samples of tumor and normal tissues from K‐Ras (+) patients, immunohistochemical analyses were performed to detect total p65/RelA and phosphorylated p65/RelA (Ser276).
**Fig. S7.** Total RNA was extracted from normal and tumor tissues from each patient harboring KRas mutations.

**Table S1.** Primers used in quantitative PCR for NF‐kB target genes.

**Table S2.** Primers used for genotyping of KRas gene.
