# Supplementary material for: Oncogenic Ras mutant causes the hyperactivation of NF‐κB via acceleration of its transcriptional activation
Source: Mol Oncol. 2019 Oct 18;13(11):2493–510. doi: 10.1002/1878-0261.12580 (PMC6822247; doi:10.1002/1878-0261.12580)
Supplement: Supplementary file 1 — Fig. S1. (A) KF‐8 cells were infected with control retroviruses or retroviruses harboring K‐Ras (G12V) or c‐Myc. After puromycin selection, cells were stimulated with 10 μg/ml TNFa for indicated periods. Then, cells were lysed, and the luciferase assay was performed. (B) Using KF‐8 cells infected with control retroviruses or retroviruses harboring K‐Ras mutants including G12V, K117N and A146T, the luciferase assay was performed as shown in (A). In the graph, error bars = SD (n = 3, *P < 0.005). Fig. S2. (A) HEK293T cells were transfected with a luciferase vector including κB‐responsive element with/without FLAG‐PKAc, a catalytic subunit of PKA. Then, using the transfected cells, luciferase assays were performed. In graph, error bars indicate standard deviation (S.D., n = 3), and the results of calculations of independent t‐tests are shown (*P < 0.005). (B) The expression of FLAG‐MSK1 and H‐Ras (G12V) in cells analyzed for in vitro kinase assay (Figure 5A) was analyzed by immunoblotting analysis using antibodies against FLAG‐tag (M2) and H‐Ras. (C) KF‐8 cells were infected with retroviruses harboring scrambled short‐hairpin RNA, sh‐MSK1 (1A or 1B) or sh‐MSK2 (2A or 2B). After puromycin selection, cells were lysed, and immunoblotting analysis was performed to evaluate the knockdown efficiency of MSK1 and MSK2. β‐actin was used as a loading control. Fig. S3. (A) NIH‐3T3 cells were infected with indicated retroviruses as described in Figure 5B. After puromycin selection, cells (1 × 10^4^ cells) were seeded on soft agar media in 35 mm dishes. After 3 weeks, the number of colonies were counted and shown in graph as shown in (B). In the graph, error bars = SD (n = 3, *P < 0.005). In the photograph, 500 μm scale bar was shown. Fig. S4. SW620, a colorectal cancer‐derived cell line was transfected with siRNAs against both MSK1 and MSK2. Fig. S5. Genomic DNA was extracted from tumor tissues of 30 colorectal cancer patients (designated as T001 to T030). Fig. S6. Utilizing paired [file MOL2-13-2493-s001.pdf]

Supplementary Figures

For:

Oncogenic Ras mutant causes the hyper-activation of NF-κB via acceleration of its transcriptional activation.

Kenji Tago[1, *], Megumi Funakoshi-Tago[2], Satoshi Ohta[1], Hirotoshi Kawata[3], Hiroshi Saitoh[1], Hisanaga Horie[4], Chihiro Aoki-Ohmura[1], Junji Yamauchi[5], Akira Tanaka[3], Jitsuhiro Matsugi[1] and Ken Yanagisawa[1]

[1] Division of Structural Biochemistry, Department of Biochemistry, [3]Department of Pathology, [4]Department of Surgery, Jichi Medical University, 3311-1 Yakushiji, Shimotsuke, Tochigi, Japan, 321-0498, [2] Division of Hygienic Chemistry, Faculty of Pharmacy, Keio University, 1-5-30 Shibakoen, Minato-ku, Tokyo 105-8512, Japan, [5] Laboratory of Molecular Neuroscience and Neurology, Tokyo University of Pharmacy and Life Sciences, Hachioji, Tokyo, 192-0392, Japan

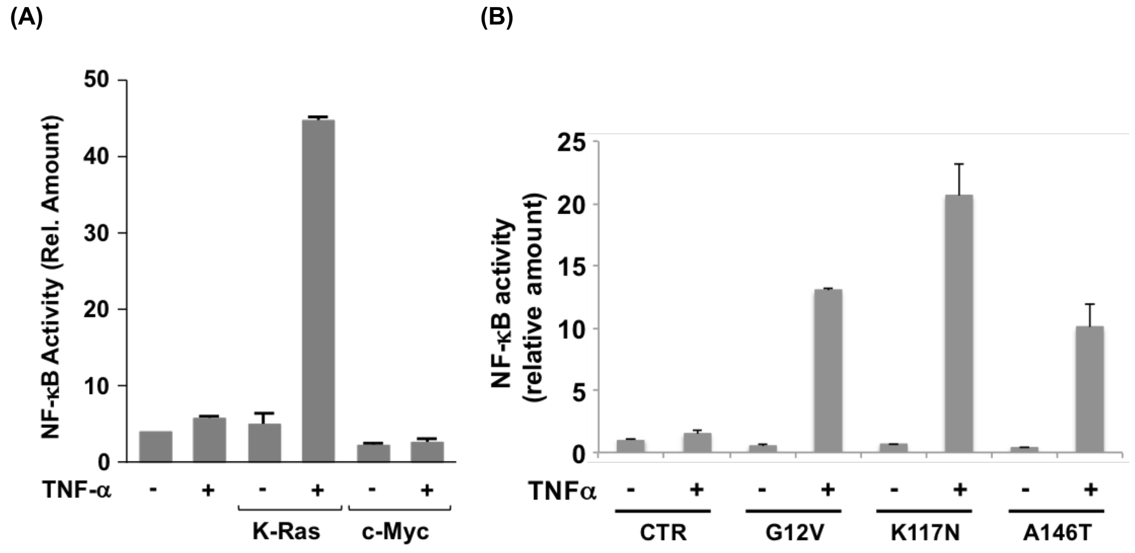

Supplementary Figure 1

(A) KF-8 cells were infected with control retroviruses or retroviruses harboring K-Ras (G12V) or c-Myc. After puromycin selection, cells were stimulated with 10 μg/ml TNFα for indicated periods. Then, cells were lysed, and the luciferase assay was performed. (B) Using KF-8 cells infected with control retroviruses or retroviruses harboring K-Ras mutants including G12V, K117N and A146T, the luciferase assay was performed as shown in (A). In the graph, error bars = S.D. (n=3, *P<0.005).

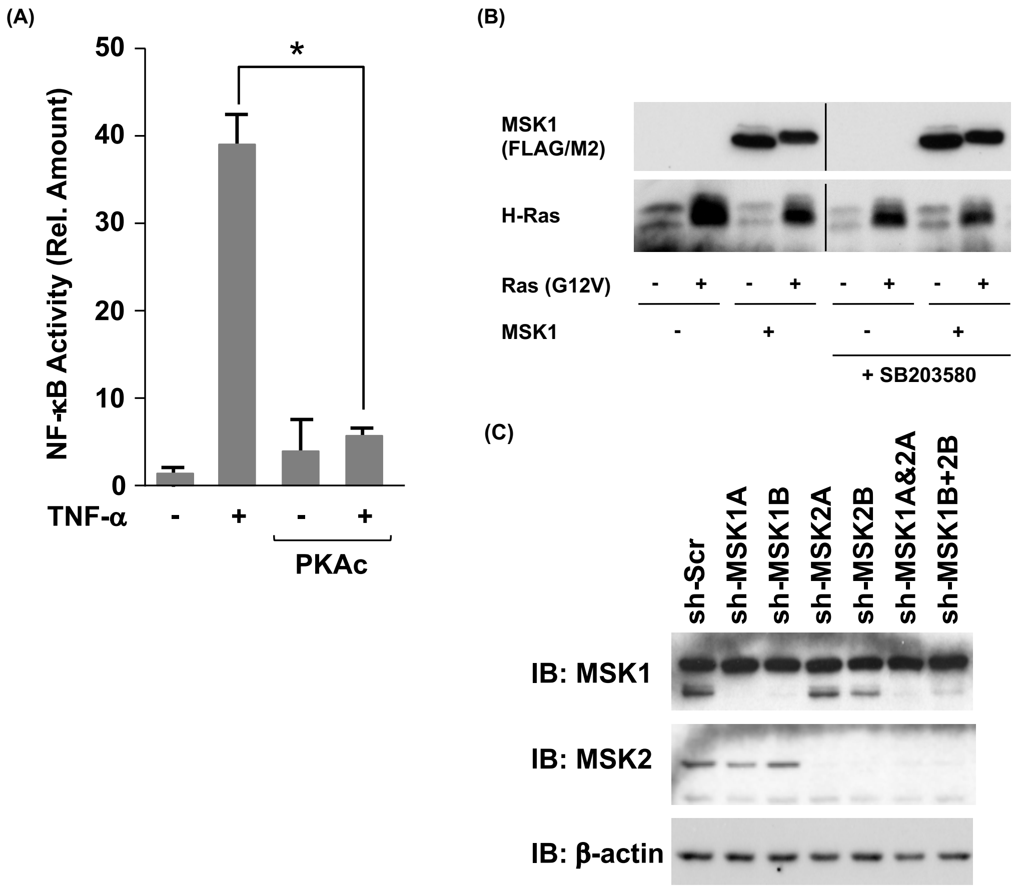

Supplementary Figure 2

(A) HEK293T cells were transfected with a luciferase vector including κB-responsive element with/without FLAG-PKAc, a catalytic subunit of PKA. Then, using the transfected cells, luciferase assays were performed. In graph, error bars indicate standard deviation (S.D., n=3), and the results of calculations of independent t-tests are shown (*P<0.005). (B) The expression of FLAG-MSK1 and H-Ras (G12V) in cells analyzed for *in vitro* kinase assay (Figure 5A) was analyzed by immunoblotting analysis using antibodies against FLAG-tag (M2) and H-Ras. (C) KF-8 cells were infected with retroviruses harboring scrambled short-hairpin RNA, sh-MSK1 (1A or 1B) or sh-MSK2 (2A or 2B). After puromycin selection, cells were lysed, and immunoblotting analysis was performed to evaluate the knockdown efficiency of MSK1 and MSK2. β-actin was used as a loading control.

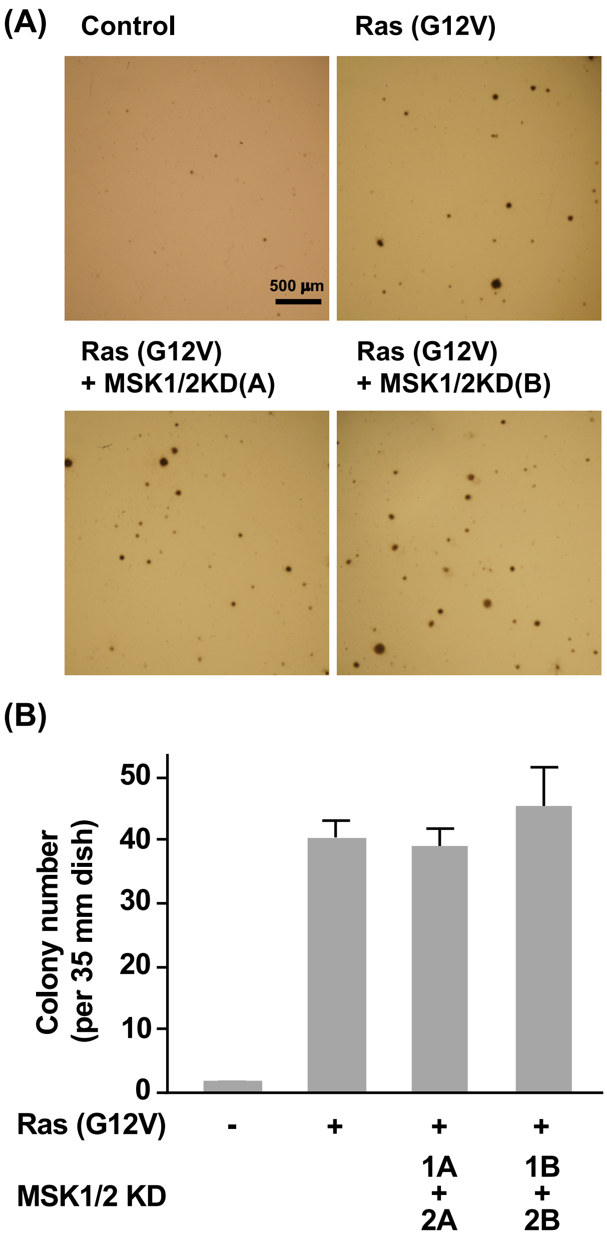

Supplementary Figure 3

(A) NIH-3T3 cells were infected with indicated retroviruses as described in Figure 5B. After puromycin selection, cells (1×10^4 cells) were seeded on soft agar media in 35 mm dishes. After 3 weeks, the number of colonies were counted and shown in graph as shown in (B). In the graph, error bars = S.D. (n=3, **P*<0.005). In the photograph, 500 µm scale bar was shown.

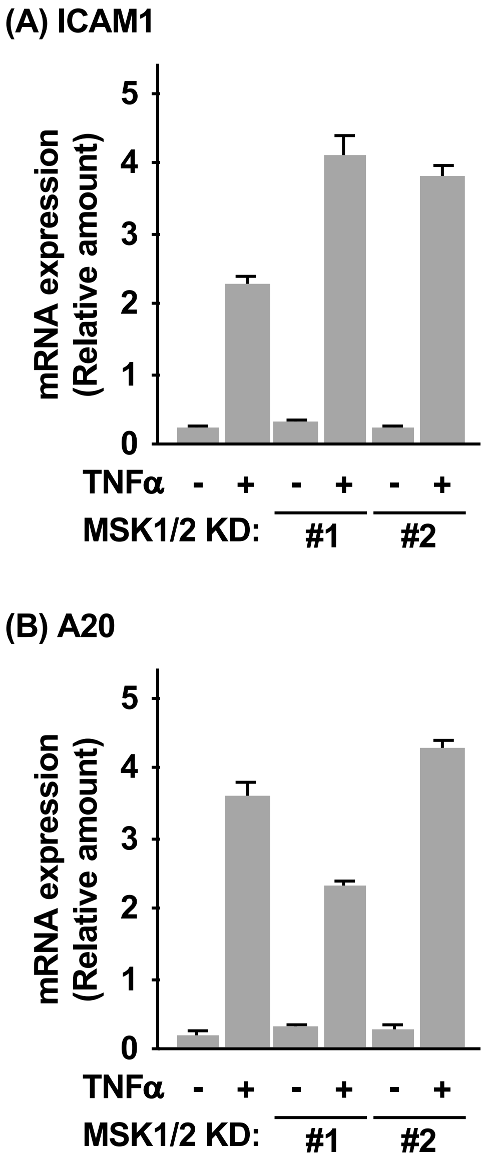

Supplementary Figure 4

SW620, a colorectal cancer-derived cell line was transfected with siRNAs against both MSK1 and MSK2. Forty-eight hr later, cells were stimulated with/without 10 ng/mL TNFα for 3 h, and then total RNAs were extracted from these cells. The expressions of NF-κB target genes, ICAM1 and A20 were analyzed by quantitative RT-PCR, and results were shown in the graphs (A) and (B). In the graph, error bars = S.D. (n=3, *P<0.005).

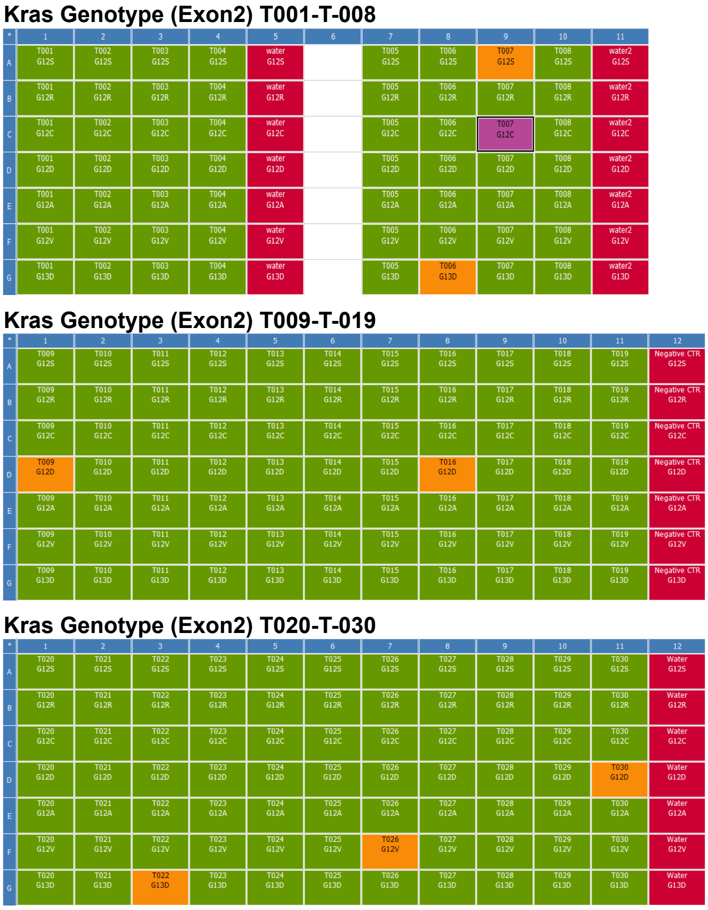

Patient Number	KRas mutation
006	G13D
007	G12S/G12C
009	G12D
016	G12D
022	G13D
026	G12V
030	G12D

Supplementary Figure 5

Genomic DNA was extracted from tumor tissues of 30 colorectal cancer patients (designated as T001 to T030). To detect K-Ras mutations such as G12S, G12R, G12C, G12D, G12A, G12V, and G13D, quantitative PCR was performed. Detected K-Ras mutations are colored purple (homozygous) or yellow (heterozygous). As result, it was concluded that 7 colorectal cancer patients (006, 007, 009, 016, 022, 026, and 030) harbored K-Ras mutations in exon 2 of the K-Ras gene.

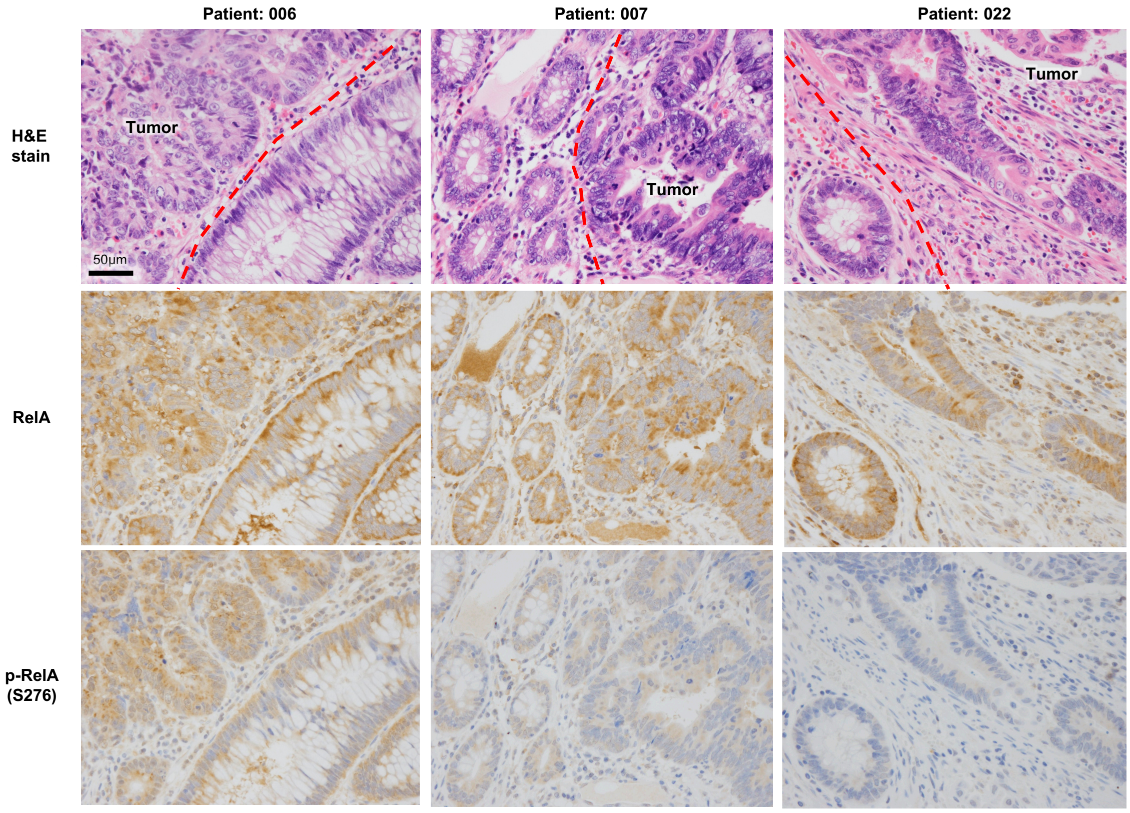

Supplementary Figure 6

Utilizing paired samples of tumor and normal tissues from K-Ras (+) patients, immunohistochemical analyses were performed to detect total p65/RelA and phosphorylated p65/RelA (Ser276). H & E staining is also shown to distinguish tumor and normal tissues. Among 7 patients, 2 samples (009 and 016) were shown in Figure 7A. In the photograph, 50 μm scale bar was shown excepting patient 026 including 100 μm scale bar.

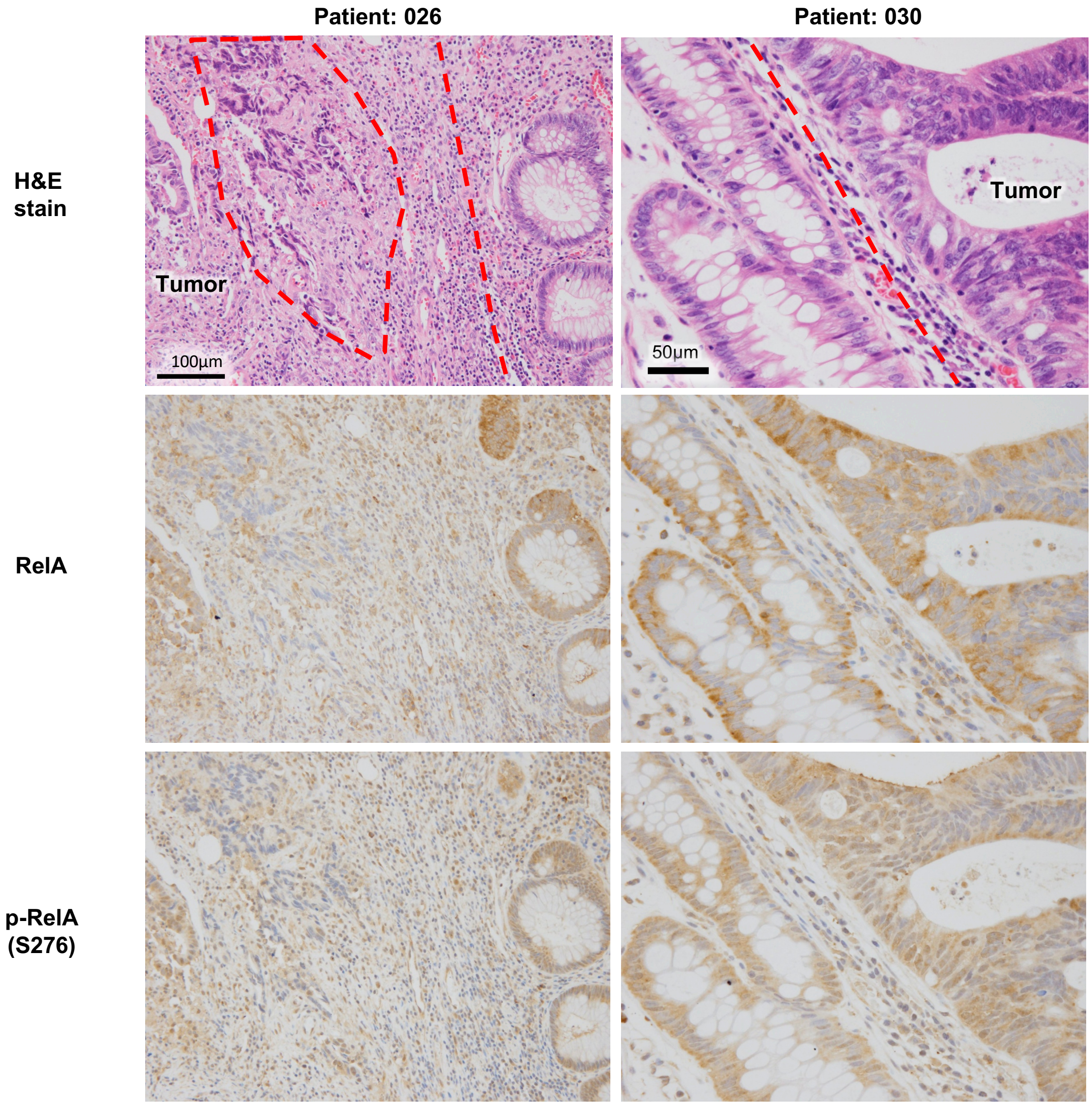

Supplementary Figure 6

(Continued)

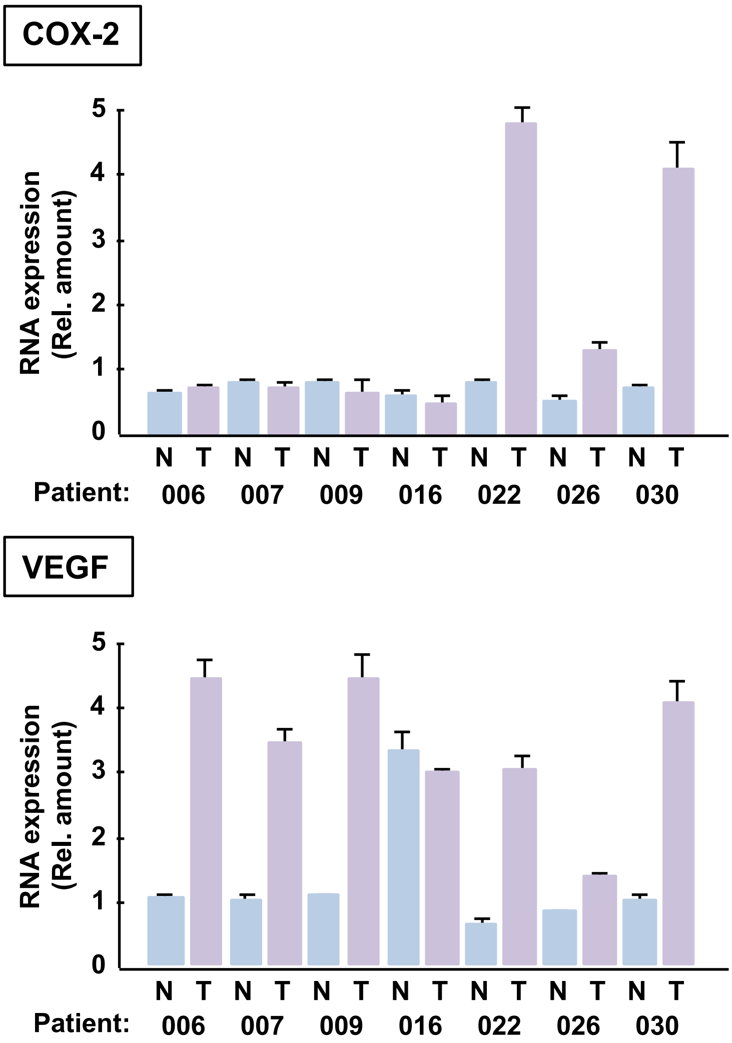

Supplementary Figure 7

Total RNA was extracted from normal and tumor tissues from each patient harboring K-Ras mutations. Then, quantitative RT-PCR was performed to evaluated the mRNA expression of COX-2 and VEGF. In the graph, error bars = S.D. (n=3).
