# Supplementary material for: Oncogenic Ras mutant causes the hyperactivation of NF‐κB via acceleration of its transcriptional activation
Source: Mol Oncol. 2019 Oct 18;13(11):2493–510. doi: 10.1002/1878-0261.12580 (PMC6822247; doi:10.1002/1878-0261.12580)
Supplement: Supplementary file 2 — Table S1. Primers used in quantitative PCR for NF‐kB target genes. [file MOL2-13-2493-s002.pdf]

Supplementary Table 1: Primers used in quantitative PCR for NF-κB target genes

Analyzed Gene	Primers	Sequences
COX2	Forward Primer	GTGCAACACTTGAGTGGCTAT
	Reverse Primer	GCAATTTGCCTGGTGAATGAT
ICAM1	Forward Primer	AAGGATGGCACTTTCCCACT
	Reverse Primer	GTGATGATGACAATCTCATACCG
A20	Forward Primer	GGGTGGAATTTACTTGCC
	Reverse Primer	AGGGTCACCAAGGGTACA
VEGF	Forward Primer	CGGCGAAGAGAAGAGACACATTG
	Reverse Primer	CGGGAAGGGAAGGGAAGGAC
Ubiquitin	Forward Primer	GGAAGGCATTCCTCCTGAT
	Reverse Primer	CCCACCTCTGAGACGGAGTA
