# Supplementary material for: Oncogenic Ras mutant causes the hyperactivation of NF‐κB via acceleration of its transcriptional activation
Source: Mol Oncol. 2019 Oct 18;13(11):2493–510. doi: 10.1002/1878-0261.12580 (PMC6822247; doi:10.1002/1878-0261.12580)
Supplement: Supplementary file 3 — Table S2. Primers used for genotyping of KRas gene. [file MOL2-13-2493-s003.pdf]

Supplementary Table 2: Primers used for genotyping of KRas gene

KRas mutation	Primer/Probe	Sequences
c.34G>A	Forward primer	TGCTGAAAATGACTGAATATAAACTTGTGG
	Reverse primer	GCTGTATCGTCAAGGCACTCTT
	Probe for WT KRas (VIC)	TTGGAGCTGGTGGCGTA
	Probe for mut KRas (FAM)	TAGTTGGAGCTAGTGGCGTA
c.34G>C	Forward primer	TGCTGAAAATGACTGAATATAAACTTGTGG
	Reverse primer	GCTGTATCGTCAAGGCACTCTT
	Probe for WT KRas (VIC)	TTGGAGCTGGTGGCGTA
	Probe for mut KRas (FAM)	TTGGAGCTCGTGGCGTA
c.34G>T	Forward primer	TGCTGAAAATGACTGAATATAAACTTGTGG
	Reverse primer	GCTGTATCGTCAAGGCACTCTT
	Probe for WT KRas (VIC)	TTGGAGCTGGTGGCGTA
	Probe for mut KRas (FAM)	TTGGAGCTTGTGGCGTA
c.35G>A	Forward primer	GCTGAAAATGACTGAATATAAACTTGTGGTA
	Reverse primer	GCTGTATCGTCAAGGCACTCTT
	Probe for WT KRas (VIC)	TTGGAGCTGGTGGCGTA
	Probe for mut KRas (FAM)	TTGGAGCTGATGGCGTA
c.35G>C	Forward primer	TGCTGAAAATGACTGAATATAAACTTGTGG
	Reverse primer	GCTGTATCGTCAAGGCACTCTT
	Probe for WT KRas (VIC)	TTGGAGCTGGTGGCGTA
	Probe for mut KRas (FAM)	TTGGAGCTGCTGGCGTA
c.35G>T	Forward primer	TGCTGAAAATGACTGAATATAAACTTGTGG
	Reverse primer	GCTGTATCGTCAAGGCACTCTT
	Probe for WT KRas (VIC)	TTGGAGCTGGTGGCGTA
	Probe for mut KRas (FAM)	TTGGAGCTGTTGGCGTA
c.38G>A	Forward primer	TGCTGAAAATGACTGAATATAAACTTGTGG
	Reverse primer	GAATTAGCTGTATCGTCAAGGCACT
	Probe for WT KRas (VIC)	CTTGCCTACGCCACCAG
	Probe for mut KRas (FAM)	CTTGCCTACGTCACCAG
